# Anonymous HIV workplace surveys as an advocacy tool for affordable private health insurance in Namibia

**DOI:** 10.1186/1758-2652-12-32

**Published:** 2009-11-11

**Authors:** Ingrid de Beer, Hannah M Coutinho, Peter J van Wyk, Esegiel Gaeb, Tobias Rinke de Wit, Michèle van Vugt

**Affiliations:** 1 PharmAccess Foundation Namibia, Windhoek, Namibia; 2 PharmAccess Foundation, Center for Poverty-related Communicable Disease, Academic Medical Center, Amsterdam, The Netherlands; 3 Namibia Business Coalition for AIDS, Windhoek, Namibia; 4 Namibia Institute of Pathology, Windhoek, Namibia; 5 Center for Poverty-related Communicable Diseases, Academic Medical Center, University of Amsterdam, Amsterdam, The Netherlands; 6 Division of Infectious Diseases, Tropical Medicine and AIDS, Academic Medical Center, University of Amsterdam, Amsterdam, The Netherlands

## Abstract

**Background:**

With an estimated adult HIV prevalence of 15%, Namibia is in need of innovative health financing strategies that can alleviate the burden on the public sector. Affordable and private health insurances were recently developed in Namibia, and they include coverage for HIV/AIDS. This article reports on the efficacy of HIV workplace surveys as a tool to increase uptake of these insurances by employees in the Namibian formal business sector. In addition, the burden of HIV among this population was examined by sector.

**Methods:**

Cross-sectional anonymous HIV prevalence surveys were conducted in 24 private companies in Namibia between November 2006 and December 2007. Non-invasive oral fluid-based HIV antibody rapid tests were used. Anonymous test results were provided to the companies in a confidential report and through presentations to their management, during which the advantages of affordable private health insurance and the available insurance products were discussed. Impact assessment was conducted in October 2008, when new health insurance uptake by these companies was evaluated.

**Results:**

Of 8500 targeted employees, 6521 were screened for HIV; mean participation rate was 78.6%. Overall 15.0% (95% CI 14.2-15.9%) of employees tested HIV positive (range 3.0-23.9% across companies). The mining sector had the highest percentage of HIV-positive employees (21.0%); the information technology (IT) sector had the lowest percentage (4.0%). Out of 6205 previously uninsured employees, 61% had enrolled in private health insurance by October 2008. The majority of these new insurances (78%) covered HIV/AIDS only.

**Conclusion:**

The proportion of HIV-positive formal sector employees in Namibia is in line with national prevalence estimates and varies widely by employment sector. Following the surveys, there was a considerable increase in private health insurance uptake. This suggests that anonymous HIV workplace surveys can serve as a tool to motivate private companies to provide health insurance to their workforce. Health insurance taken up by those who are able to pay the fees will alleviate the burden on the public sector.

## Background

HIV predominantly affects adults of working age. On a global scale, the majority of these adults live in sub-Saharan Africa [1], where Namibia is among the countries hardest hit by the epidemic. According to most recent estimates, adult HIV prevalence in Namibia is 15.3%, with a plausibility range of 12.4-18.1% [2].

Large-scale implementation of highly active antiretroviral treatment (HAART) in sub-Saharan Africa is currently taking place. An estimated 2.1 million people in this region are now receiving antiretroviral treatment under World Health Organization (WHO) guidelines, which comes down to approximately one out of every three HIV-infected people in need of treatment [3]. As a consequence, analogous to developments in the western world after the introduction of HAART, a shift towards HIV/AIDS as a chronic disease is taking place in the region, with opportunistic infections and co-morbidity becoming increasingly important [3].

The life-long quality care and treatment that is required for the masses of HIV-infected patients will further increase the demands placed on the already overburdened and understaffed public health care systems in sub-Saharan Africa. Notwithstanding the extraordinary global surge in funding, the financial costs of the HIV/AIDS epidemic are expected to rise more than four-fold if prevention and treatment scale up continues at the same pace as today [4].

Moreover, the region is facing a general transition in health challenges, with chronic non-communicable diseases, such as diabetes and cardiovascular diseases, taking over from infectious diseases as the most important cause of morbidity and mortality [5]. Because chronic diseases are more expensive to treat and cause long-term disability, the demands on health care infrastructure and capacity are expected to further increase [5].

The current HIV-1 prevention and treatment strategies in sub-Saharan Africa are largely being implemented through civil society and the public sector. Although the private business sector is affected by the epidemic [6] and workplace programmes were the first to pioneer HIV-1 treatment in the region [7,8], public HIV/AIDS treatment programmes have largely taken over, supported by large international funds [4]. Today, only a limited number of multinationals and an even smaller number of small and medium enterprises (SMEs) offer an HIV/AIDS programme to their employees [9-11]. Approximately 26% of the sub-Saharan African companies that have HIV policies provide antiretroviral treatment to their workers [7].

Sustainability of public HIV/AIDS prevention and treatment programmes in the long run is questionable given their heavy reliance on donor funds. In addition, the necessity to integrate these programmes into existing primary health care systems and improve the efficacy of these systems will greatly increase the costs, logistical challenges and required human resources [3]. Additional, complementary approaches, such as health insurance, are therefore required to enable the long-term success of global efforts to improve health care in developing countries.

Major benefits of health insurance include protection of individuals against catastrophic health expenditures, increased solidarity through financial risk pooling, and the possibility to channel "vertical" funds, such as for HIV/AIDS, into general health financing [12,13]. Currently, the majority of those with access to health insurance in sub-Saharan Africa are the urban elite, in particular higher income formal sector workers, who can obtain coverage (partly) subsidized through their employers [9,13].

In Namibia, approximately 12.5% of the population was covered by health insurance in 2004 [14]. PharmAccess Foundation, a not-for-profit organization that aims to improve access to affordable and sustainable quality health care provision in sub-Saharan Africa, supported the launch of several Namibian health insurance packages aimed at low- and middle-income workers. Crucial in this was the development of a risk equalisation fund for HIV/AIDS (HIVREF) in 2006, which enabled individual health insurance providers to share the risks for this disease. Thus, otherwise competing health insurers can collaborate in this unique solidarity fund [9].

As a special option, employer and/or employee groups that cannot afford the primary health insurance can purchase an "HIV/AIDS only" package, covered by the HIVREF. This HIV/AIDS health insurance is compulsory for all employees of a company that decides to enrol, while enrolment in the majority of primary health insurances is voluntary.

PharmAccess Foundation recently conducted several anonymous HIV workplace surveys in the formal business sector in Namibia with the aim of stimulating employers to provide the affordable health insurance products that we have described to their employees. It was hypothesized that providing companies with HIV prevalence estimates of their workforces would create awareness among the management and thereby lead to health insurance uptake. This article reports the results of these surveys and is the first quantitative documentation of the burden of HIV among employees in the Namibian formal sector.

## Methods

### Survey design and implementation

Between November 2006 and December 2007, cross-sectional anonymous HIV surveys were conducted among employees of 24 private companies throughout Namibia. The surveys were conducted by PharmAccess Foundation Namibia, in partnership with the Namibia Business Coalition on AIDS (NABCOA) and the Namibia Institute of Pathology.

NABCOA was launched in 2003 to mobilize the private business sector in the national HIV/AIDS response [15]; it did so through its "Healthy Workforce, Healthy Business" programme. Companies that expressed interest in HIV prevalence surveillance following this programme were referred to PharmAccess for implementation of HIV workplace surveys. The major incentive for companies to participate in these surveys was to obtain information to develop or improve HIV/AIDS workplace programmes.

In each company, surveys were prepared and conducted as follows. First, awareness-raising presentations were provided to the management, which stressed the value of HIV prevalence estimates for internal HIV/AIDS policy. In addition, indirect effects of the surveys, such as increased awareness about HIV/AIDS among employees, were discussed. Second, education and sensitization sessions were held for both management and employees on the process of surveillance and the importance of participation. During these sessions, the importance of access to treatment and the need to mitigate the impact of HIV on the business was highlighted. The availability of affordable health insurance packages was introduced as a risk-mitigation intervention. Third, anonymous and voluntary HIV prevalence surveys were conducted. Finally, anonymous survey results were presented to the management and advocacy meetings were held to stimulate company uptake of affordable private health insurance, including HIV/AIDS coverage, for employees.

### HIV testing and confidentiality

For HIV testing, OraQuick Rapid HIV-1/2 Antibody Tests (OraSure Technologies, Inc, Bethlehem, PA ["OraQuick"]) were used. This non-invasive HIV rapid test was validated in Namibian high-risk populations in 2005, showing 100% sensitivity and 100% specificity [16].

Because HIV results of the survey were not disclosed on an individual level, all participating employees were encouraged to visit a voluntary counselling and testing facility to obtain their HIV status in accordance with national HIV testing requirements. To guarantee confidentiality and ensure willingness to participate among employees, collection of demographic data was limited and included only sex and age. Data on age were collected either in exact years or in age categories, depending on the size of the company, to ensure confidentiality and encourage maximum participation.

### Impact assessment on health insurance uptake

It was hypothesized that providing companies with HIV prevalence estimates of their workforce would create awareness among the management and thereby lead to health insurance uptake. To test this hypothesis, impact assessment was conducted as follows. In October 2008, when all cross-sectional HIV workplace surveys had been conducted, the number of new insurance policies taken up by employees after the survey had been conducted was reviewed. Data were obtained from several databases that record data on insurance policies of the main providers in Namibia. PharmAccess has access to these databases as part of its external quality control responsibilities. Information on uptake of insurances that were not recorded in this database was obtained directly from the companies.

Statistical analyses

Statistical analyses were performed with SPSS version 15.0 for Windows, Chicago: SPSS Inc. For significance testing, Chi square and Student's T-test were used for dichotomous and continuous variables, respectively. *P*-values < 0.05 were considered statistically significant.

## Results

### HIV test results

Table 1 shows overall HIV results of the surveys, stratified by industry and company, as well as by new insurance uptake. Overall, 6521 of 8500 targeted employees participated in the HIV surveys in 24 companies located throughout Namibia. Participation rates within companies varied from 61.3% to 97.3%, with a mean (95% CI) participation rate of 78.6% (78.3-78.8%). In total 980 out of 6521 employees tested HIV positive, suggesting an HIV prevalence of 15.0% (95% CI 14.2-15.9%). This proportion varied from 3.0-23.9% between companies (Table 1).

**Table 1:** HIV results by company and new insurances taken up by October 2008

*Industry*	*Company*	*Participation rate* ^1^		*Participation by sex (M/F)* ^2^	*HIV positive*		*New insurances* ^3^		*Insurance type* ^4^
		**No.**	**%**	**No.**	**No.**	**%**	**No.**	**%**	
**Transport**	1	308/447	68.9	132/176	49	15.9	113	25.3	Traditional
**Tourism**	2	165/239	69.0	-	26	15.8	178	74.5	HIV only
	3	127/149	85.2	-	6	4.7	118	79.2	HIV only
**Retail**	4	714/863	82.7	-	77	10.8	578	70	HIV only
**Manufacturing**	5	349/425	82.1	-	53	15.2	297	69.9	HIV only
	6	511/525	97.3	-	54	10.6	359	68.4	HIV only
	7	88/105	83.8	-	21	23.9	87	82.9	HIV only
	8	202/215	94.0	149/53	29	14.4	98	45.6	HIV only
	9	248/296	83.8	205/43	52	21.0	289	95.7	HIV only
	10	400/653	61.3	332/68	88	22.0	924	145.5 ^7^	HIV only
**Wholesale**	11	115/137	83.9	54/61	9	7.8	18 ^9^	13.1	Traditional
	12	54/61	88.5	35/19	3	5.6	4 ^9^	6.6	Traditional
**IT**	13	25/31	80.6	-	1	4.0	12 ^8^	12.1	HIV only
**Services**	14	74/92	80.4	-	4	5.4			
	15	77/82	93.9	-	18	23.4	68	82.9	HIV only
	16	383/625	61.3	311/72	65	17.0	324	51.8	Affordable
	17	235/319	73.7	112/123	7	3.0	7	2.2	Traditional
	18	102/131	77.9	86/16	8	8.8	0	0	-
	19	155/161 ^5^	96.3	44/111	5	3.2	3 ^9^	1.9	Traditional
**Financial services**	20	279/374	74.6	128/151	39	14.0	0 ^9^	0	-
**Fishing**	21	664/1049	63.3	-	121	18.2	774	73.8	HIV only
	22	287/435 ^6^	66.0	86/201	49	17.1	328	75.4	HIV only
**Agriculture**	23	154/177	87.0	-	26	16.9	153	86.4	HIV only
**Mining**	24	805/909	88.7	724/82	169	21.0	47 ^9^	5.2	Traditional

Total or mean	**N = 24**	**6521/8500**	**78.6**	**2398/1175**	**980**	**15.0**	**4779**	**56.2**	**-**

^1 ^Participation rate is defined as number of participating employees relative to target population. Target population is defined as total number of employees within company at time of workplace survey

^2 ^Sex was recorded for 3573 of 6521 (54.8%) participating employees

^3 ^Percentage of new insurances is defined as number of new insurances relative to the total number of employees per company at the time of the survey

^4 ^Traditional insurance, which existed prior to introducing affordable insurance products, entails income dependent individual monthly premiums of N$800-2300; for affordable insurance, the age dependent monthly premium is N$250-350; for HIV coverage only, the monthly premium for all is N$30

^5 ^N = 155/161 (96.3%) employees were on site on the day the survey was performed; use of this number would result in participation rate of 100%

^6 ^N = 292/435 (67.1%) employees were on site on the day the survey was performed; use of this number would result in participation rate of 98.3%

^7 ^Temporary employees, who were not part of the survey, were included in the new insurances taken up by this company

^8 ^Insurance data of companies 13 and 14 could not be evaluated separately and were thus combined

^9 ^All employees were insured at the time of the workplace survey

**Figure 1:**
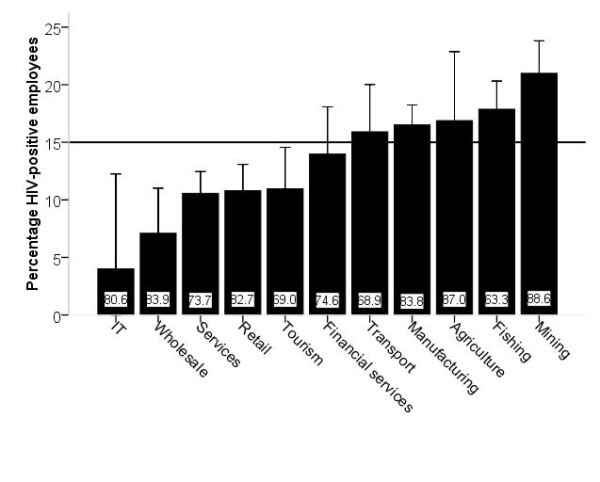
**Proportion of HIV-positive employees stratified by industry**. Numbers at bottom of bars represent mean participation rate per industry category. Error bars represent 95% confidence intervals. The horizontal line represents mean percentage of HIV-positive employees in the entire cohort.

Figure 1 shows the proportion of employees who tested HIV positive, stratified by employment industry. Transport, manufacturing, agriculture, fishing and mining appear to be "high-risk industries", defined as those with a proportion of HIV-positive employees greater than the overall survey mean of 15.0%. The mining sector had the highest proportion of HIV-positive employees (21.0%), whereas this was lowest in the information technology sector (4.0%). However, in the latter sector, only a small number of employees were tested.

In order to guarantee confidentiality, data on sex were not collected in 11 of the 24 companies, resulting in registered sex for 3572 (54.8%) employees. In the 13 companies where sex was registered, the majority of participants were male (67.1%; between company range of 28.4-89.9%; Table 1). In all, 431 of 2394 men (18.0%; 95% CI 16.5-19.6%) and 142 of 1175 women (12.1%; 95% CI 10.3-14.0%) tested HIV positive (*p *< 0.0001).

**Figure 2:**
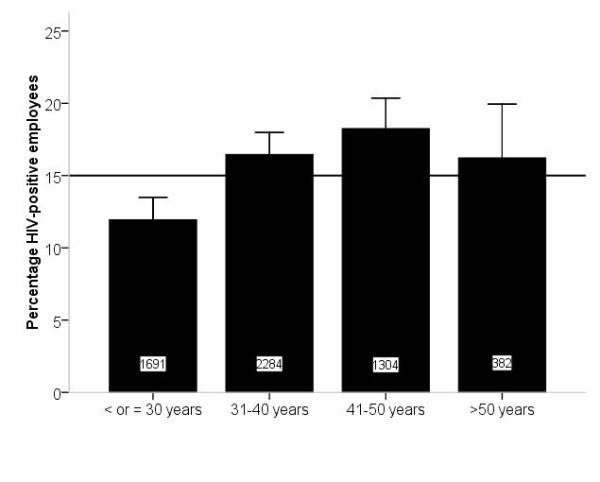
**Proportion of HIV-positive employees stratified by age**. Data shown represent 86.8% of the cohort. Numbers at the bottom of the bars represent total number of tested individuals per age category. Error bars represent 95% confidence intervals. The horizontal line represents mean percentage of HIV-positive employees in the entire cohort.

Age was registered for 6514 (99.9%) employees. Exact age was registered for 2718 (41.7%) employees in nine of 24 companies; in this subgroup, mean age (95% CI) was 35.1 (34.8-35.5) years, with a range of 18 to 69 years. Mean age (95% CI) among employees who tested HIV positive and negative was 36.1 (35.2-36.9) and 35.0 (34.5-35.4) years, respectively (*p *= 0.03). For the remaining 3796 employees, age was registered in categories. HIV distribution by age is shown in Figure 2. Individuals in their 40 s had the highest risk to test HIV positive, whereas those younger than 30 years old had the lowest risk. Sex-stratified analysis showed an equal HIV distribution for women across age categories; the range of women testing HIV positive was 13.5-14.6% across age categories. Results were quite different for men. In the 31-50 years age group, 23% of men tested HIV positive, versus 15% in men younger than 31 or older than 50 years. Of note, these estimates are based on a relatively small proportion of the cohort (27.7%) because of the large amount of missing data on sex.

### Impact assessment on health insurance uptake

It was hypothesized that HIV workplace surveys would result in increased uptake of affordable private health insurance by formal sector employees. In October 2008, which was between 10 and 21 months after the surveys had been conducted in the 24 companies, 4779 new insurances were registered (Table 1). This comes down to coverage of 56% of the employees working at one of these companies at the time of the survey, assuming a constant workforce. The broad range of new insurances, varying from 0-146% between companies, can be explained in part by the fact that five of the 24 companies already provided health insurance to all their employees at the time of the survey. In addition, one company provided insurance to both permanent and temporary employees; the latter group did not participate in any of the HIV surveys (Table 1). Exclusion of these six companies resulted in 3783 new insurances in the remaining 18 companies, which employed 6205 individuals at the time of the survey. This suggests that 61% of the previously uninsured workforce was insured in October 2008, assuming a stable workforce. The majority (78%) of the new insurance products offered by these companies covered HIV/AIDS only. Subsidization by employers ranged from 50% to 100% of the monthly premium for the newly purchased private health insurances.

## Discussion

This study describes results of anonymous HIV workplace surveys among employees of 24 private companies in Namibia. The primary aims were to: (1) estimate HIV prevalence among formal sector employees; and (2) use these prevalence estimates as a tool to advocate implementation of affordable health insurance for employees, including HIV/AIDS coverage.

Our finding that 15% of employees tested HIV positive is in line with national prevalence estimates [2], despite the fact that formal sector employees are not a representative sample of the general population. Interestingly, among workplace survey participants whose sex was registered, men were 1.5 times more likely than women to test HIV positive. This finding contrasts with national and international HIV prevalence data in general populations, where women are generally infected at higher rates [17]. Perhaps formal sector employment, and thus increased financial independence, is a protective factor against HIV/AIDS for women. However, this finding may be biased by the large amount of missing data on sex.

Impact assessment showed that new health insurance uptake was considerable, which suggests that anonymous HIV workplace surveys can trigger implementation of private health insurance in the Namibian formal sector. After presentation of survey results to the company management, 18 of the 19 companies that did not yet provide health insurance for employees expressed a willingness to do so.

Upon evaluation in October 2008, which was 10 to 21 months after the surveys were conducted, 61% of previously uninsured employees were enrolled in private health insurance. Because we had no access to registration data of some Namibian health insurance providers, this figure is likely to be an underestimation of the number of newly purchased insurances. The current data demonstrate that even SMEs can be persuaded to invest in healthcare solutions for their workforces, despite their limited resources compared to large companies [11].

Implementation of affordable health insurance in the private business sector, including HIV/AIDS coverage, is relevant for several reasons. First, approximately 5% of health services in Namibia are currently delivered through the private health sector [15]. Through implementation and expansion of affordable private health insurance linked to output-based contracts with the private health sector, this underutilization can be improved. This will alleviate the burden currently placed on public health programmes [9,13]. These public programmes can subsequently focus their resources on the poorest segments of the population, which are unable to pay for health insurance.

Second, raising HIV awareness and knowledge remains important considering the large amount of stigma that remains a major issue in many sub-Saharan African societies [18]. By offering health insurance that covers HIV, companies may be able to promote more openness about this disease among employees.

Third, HIV infection appears more concentrated among the employed and more mobile members of society [17,19]. A household survey performed in Windhoek, Namibia's capital city, in 2006 to evaluate the effect of affordable health insurance on the population level found that the relative risk to test HIV positive for employed versus unemployed adults aged 15 to 49 years was 1.5 [20]. Moreover, HIV can be regarded as an occupational health hazard in certain employment sectors, for example, in the mining sector, where this increased risk is related to the large number of migrant workers [21].

Targeting such high-risk populations will not only serve public health needs, but also result in a healthier workforce and subsequently lead to greater productivity, a reduced need for worker replacement [6,19,22,23] and direct financial gains for the private business sector. To overcome the notion among SME managers that HIV/AIDS is not a relevant problem among their workforces [11], anonymous HIV workplace surveys can aid in creating awareness and making informed decisions.

Limitations of this study need to be discussed. First, we were unable to directly measure an impact of our surveys on health insurance status of employees. Data on the number of insured employees prior to conducting the surveys, or insurance premium subsidization by employers following the surveys, could not be collected due to the operational nature of our research. Instead, we used an overview of newly registered insurances of the main insurance companies as a proxy for employee insurance status several months after conducting the surveys. This indirect impact assessment assumed that the workforce of the companies remained constant, since we were unable to obtain data on employee turnover.

Nevertheless, the considerable increase in new insurance uptake by employees does suggest our surveys may have triggered this. Prior to the surveys, none of the companies offered insurance covering HIV/AIDS only, which was taken up by the majority of employees. In addition, to the best of our knowledge, there were no targeted marketing campaigns by insurance companies following our surveys.

Second, because self-selection following the NABCOA campaign was the reason for companies to participate in our surveys, participation bias cannot be excluded. Finally, health care requirements of HIV/AIDS-related morbidity in sub-Saharan Africa have become more complex and demanding since large-scale treatment has become available. Insurance products that focus on HIV/AIDS only are therefore outdated. In Namibia, we are currently piloting "wellness workplace surveys" that focus on both HIV/AIDS and chronic diseases, such as diabetes and cardiovascular diseases. These surveys may motivate private companies to provide health insurance products with more extensive coverage to their workforces, in particular with respect to chronic diseases that require lifelong treatment.

## Conclusion

In conclusion, this study describes results from the largest workplace-based HIV survey performed in Namibia to date. The proportion of HIV-positive formal sector employees is in line with national prevalence estimates and varies widely by employment sector. The considerable increase in health insurance uptake suggests that anonymous HIV workplace surveys can serve as a tool to implement private health insurance in the formal business sector.

To sustain current HIV/AIDS prevention and treatment strategies in developing countries, cooperation of private and public efforts is required. Private health insurance, paid by those who can afford the premiums, can alleviate the burden on the public health system [9] and thereby make an important contribution to sustainable health care systems in the developing world.

## Competing interests

The authors declare that they have no competing interests.

## Authors' contributions

IDB conceived the project, collected data and edited the manuscript. HMC analyzed the data and wrote the manuscript. PJW conceived the project and edited the manuscript. EG was responsible for the HIV test results and edited the manuscript. TRW conceived the project and supervised and edited the manuscript. MVV conceived the project and supervised and edited the manuscript. All authors gave final approval of the version to be published.
